# Novel insights into the pathways regulating the canine hair cycle and their deregulation in alopecia X

**DOI:** 10.1371/journal.pone.0186469

**Published:** 2017-10-24

**Authors:** Magdalena A. T. Brunner, Vidhya Jagannathan, Dominik P. Waluk, Petra Roosje, Monika Linek, Lucia Panakova, Tosso Leeb, Dominique J. Wiener, Monika M. Welle

**Affiliations:** 1 Institute of Animal Pathology, Vetsuisse Faculty, University of Bern, Bern, Switzerland; 2 DermFocus, University of Bern, Bern, Switzerland; 3 Institute of Genetics, Vetsuisse Faculty, University of Bern, Bern, Switzerland; 4 Department of Clinical Research, Molecular Dermatology and Stem Cell Research, University of Bern, Bern, Switzerland; 5 Division of Clinical Dermatology, Vetsuisse Faculty, University of Bern, Bern, Switzerland; 6 AniCura Tierärztliche Spezialisten, Hamburg, Germany; 7 Clinics of Small Animals and Horses, University of Veterinary Medicine, Vienna, Austria; University of Pisa, ITALY

## Abstract

Alopecia X is a hair cycle arrest disorder in Pomeranians. Histologically, kenogen and telogen hair follicles predominate, whereas anagen follicles are sparse. The induction of anagen relies on the activation of hair follicle stem cells and their subsequent proliferation and differentiation. Stem cell function depends on finely tuned interactions of signaling molecules and transcription factors, which are not well defined in dogs. We performed transcriptome profiling on skin biopsies to analyze altered molecular pathways in alopecia X. Biopsies from five affected and four non-affected Pomeranians were investigated. Differential gene expression revealed a downregulation of key regulator genes of the Wnt (*CTNNB1*, *LEF1*, *TCF3*, *WNT10B*) and Shh (*SHH*, *GLI1*, *SMO*, *PTCH2*) pathways. In mice it has been shown that Wnt and Shh signaling results in stem cell activation and differentiation Thus our findings are in line with the lack of anagen hair follicles in dogs with Alopecia X. We also observed a significant downregulation of the stem cell markers *SOX9*, *LHX2*, *LGR5*, *TCF7L1* and *GLI1* whereas *NFATc1*, a quiescence marker, was upregulated in alopecia X. Moreover, genes coding for enzymes directly involved in the sex hormone metabolism (*CYP1A1*, *CYP1B1*, *HSD17B14*) were differentially regulated in alopecia X. These findings are in agreement with the so far proposed but not yet proven deregulation of the sex hormone metabolism in this disease.

## Introduction

Hair loss, hypotrichosis or alopecia in human beings and dogs is common and possible underlying causes are numerous. An intact hair coat is maintained by lifelong cycling of the hair follicles (HFs) through periodic stages of growth (anagen), regression (catagen), and relative quiescence (telogen) [1, 2]. A fourth cycle stage, during which the club hair is shed is known as exogen [3]. Kenogen is a term for HFs, which have passed the telogen stage, lost their hair fiber (exogen) and remain empty for a certain time before a new anagen phase is initiated [4]. To sustain this cyclic regeneration, each HF relies on its epithelial stem cells (SCs) which act as a regeneration pool for the cells needed to maintain and remodel the epithelium of the continuously cycling HF. The HF SC populations are characterized by different markers and reside in niches that provide spatially distinct microenvironments for their maintenance and function [5–7].

The coordination of the HC phases and the SC activity is dependent on complex interactions between signals of the follicular (niche components) and dermal microenvironment (e.g. fibroblasts, adipocytes, immune cells, nerve fibers), systemic factors (e.g. hormones, genetics, age) and environmental factors (e.g. day light, nutrition, circadian rhythm) [8, 9]. In the mouse the signaling crosstalk between SCs and their niche has been well characterized by now. The state of the murine HF is mediated by a complex, delicately balanced interplay between ligands, their receptors and transcription factors implicated in several mutually interacting signaling pathways. These pathways include Sonic hedgehog (Shh), the wingless-type mouse mammary tumor virus integration site (Wnt)/β-catenin, transforming growth factor (Tgf)-β, fibroblast growth factor (Fgf), bone morphogenic protein (Bmp) and Notch signaling (reviewed in [7, 10]). However, despite extensive research the complete understanding how molecules of these pathways interact with each other and with other elements (e.g. pH) of the SC niche, as well as with systemic and environmental factors is still not complete. In general it can be noted that Bmp signals derived from dermal, adipose and epithelial tissue repress SC activation and proliferation, while Wnt and Shh signals initiate and promote a new HC and thus regenerate a new hair shaft.

Alopecia X (AX) is a non-inflammatory hair cycle disorder affecting most commonly Pomeranians. Different disease names were used for AX in the past, such as adult-onset hyposomatotropism, growth hormone-responsive alopecia, pseudo-Cushing’s disease, castration-responsive alopecia, biopsy-responsive alopecia, adrenal hyperplasia-like syndrome, reflecting the yet unknown pathomechanism of the disease (reviewed in [11–13]). The strong predisposition of the disease for breeds with a plush undercoat, pedigree analysis of affected dogs, and the onset of the disease at a relatively young age suggest a hereditary background [14, 15]. Initially an adrenal steroid hormone imbalance similar to the congenital adrenal hyperplasia-like syndrome, caused by a mutation in the *CYP21A2* affecting the steroid 21-hydroxylase, in humans was suggested as underlying cause [16, 17]. However, a genetic variant in this gene as the cause for AX in dogs was excluded [18] and to date the mode of inheritance and the underlying pathomechanism of the impaired hair growth remain to be elucidated. Clinically the dogs develop truncal alopecia and hyperpigmentation of the skin. Systemic illness is not associated with this disease [11, 16]. Histologically, kenogen and telogen hair follicles (HFs) predominate, whereas anagen follicles are sparse [19].

The aims of the present study were 1) to identify differentially expressed genes and pathways in the skin of Pomeranians with AX as compared to healthy control Pomeranians by transcriptome profiling and 2) to define genes which may be specifically involved in the pathogenesis of AX.

## Material and methods

### Ethics statement

All biopsies were taken with informed owner consent after sedation and/or local anesthesia with permission of the cantonal animal welfare committee, switzerland (permission number BE31/13).

### Samples

Skin biopsies were taken from 5 Pomeranians with AX (2 intact males, 2 castrated males and 1 castrated female; dogs between 2 and 7 years of age) and 4 healthy control Pomeranians (2 intact males, 1 castrated male and 1 castrated female; dogs between 1 and 12 years of age). The diagnosis was based on the clinical phenotype of the dogs, assessed by board certified veterinary dermatologists and confirmed by the histological findings. Endocrinopathies were excluded by the absence of systemic signs and appropriate laboratory tests.

From each dog with AX two 6mm punch biopsies from alopecic skin and from each control dog two 6mm punch biopsies from haired skin were taken. Both biopsies were taken in close vicinity to each other from the lateral caudal thorax in the control and alopecic dogs.

From each dog one biopsy was stored in RNA*later* (76106; Qiagen; Hombrechtikon; Switzerland) at -80°C prior to RNA extraction. The other biopsy was fixed in 10% buffered formalin, embedded in paraffin, cut as 4μm section and stained with hematoxylin and eosin prior to the histological evaluation.

### RNA extraction and cDNA sequencing

Prior to RNA extraction the skin biopsies were homogenized mechanically with the TissueRuptor device from Qiagen. Total RNA was extracted from the homogenized tissue using the RNeasy Fibrous Tissue Mini Kit (74704; Qiagen; Hombrechtikon; Switzerland) according to the manufacturer’s instructions. RNA quality was assessed with a Bioanalyzer (Agilent 2100; Agilent Technologies). From each biopsy 1μg of high quality RNA (RNA integrity number: RIN > 9) was used for stranded, paired-end cDNA library preparation (TruSeq RNA Library Prep Kit v2, Illumina). Multiplexed total cDNA libraries were sequenced on one lane using the Illumina HiSeq3000 with 2x150 bp paired-end sequencing cycles. Twenty million read pairs per stranded library were collected on average. The Illumina BCL output files with base calls and qualities were converted into FASTQ file format and demultiplexed. Data are available from the European Nucleotide Archive and GenBank (accession no. PRJEB21761). All other data are available in the paper and its Supporting Information files.

### Mapping to reference genome

All reads that passed quality control were mapped to the dog genome reference (Can.Fam3.1) by STAR aligner version 2.5.3a [20]. Reads were aligned using the following parameters: --outFilterType BySJout --outFilterMultimapNmax 50 --alignSJoverhangMin 1 --outFilterMismatchNmax 2 --outFilterMismatchNoverLmax 0.04 --alignIntronMin 20 --alignIntronMax 1000000 --alignMatesGapMax 1000000. The alignment of RNA-seq reads from each sample was summarized by the number of uniquely mapped reads per sample including both singleton and both-ends mapped and number of splice alignments per sample. The read abundance was calculated using HTseq and a NCBI transcript database (version 104) derived from the CanFam3.1 dog genome assembly [21].

### Differential expression

We used the DESeq2 package [22] to read the HTseq count data and filter for low/non-expressed genes where the count is zero in all samples and a single count in one sample. The count data were subjected to a regularized-logarithm transformation and a principal component analysis (PCA) was performed to visualize the clustering of the case and control groups. Following PCA analysis, we used DESeq2 v.1.6.3 to assess differential expression between groups. DESeq2 applies a generalized linear model (GLM) to count data assuming a negative binomial distribution. For each gene, read counts were fit to a GLM with design model (~sex + condition) where condition was the factor of interest with two states: control and AX. Transcripts were considered to be differentially expressed with a Benjamini and Hochberg false discovery rate (FDR) of < 0.01.

### Pathway analysis

The differentially expressed genes were mapped to biological networks using a manually curated proprietary database (MetaCore^™^, GeneGo) and the MetaCore pathway analysis software. For the enrichment analysis, the gene symbols of the differentially expressed proteins were uploaded into the database, and the uploaded files were matched with known genes with functional ontologies (GO) in MetaCore^™^ [23]. In addition the Panther Classification System was used for the enrichment analysis [24].

## Results

### Differentially expressed genes

**Fig 1:**
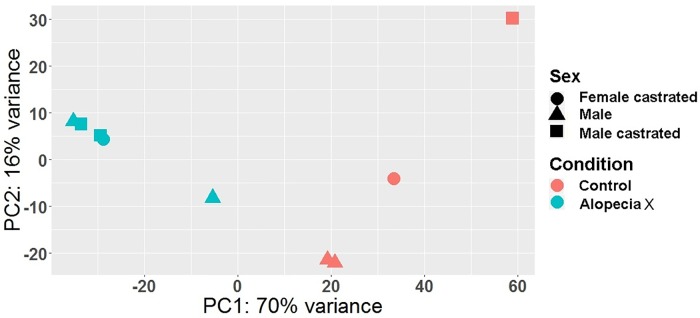
Principal component analysis of the samples in the first two component space. Samples are plotted across the two most variable components (PC1 and PC2); sample clustering is rather based on condition and not sex.

Fig 1 shows the clustering of samples based on their expression profiles. The PCA clusterization successfully separates “Alopecia X” samples from “Control” ones and is not affected by sex of the animals. Subsequently we applied the GLM model to identify differentially expressed genes between “Alopecia X” and “Control”. We identified a total of 1598 differentially expressed genes with a FDR of < 0.01. Of those, 569 genes were upregulated and 1029 were downregulated in dogs with AX (S1 Table).

### Functional classification of differentially expressed genes

**Fig 2:**
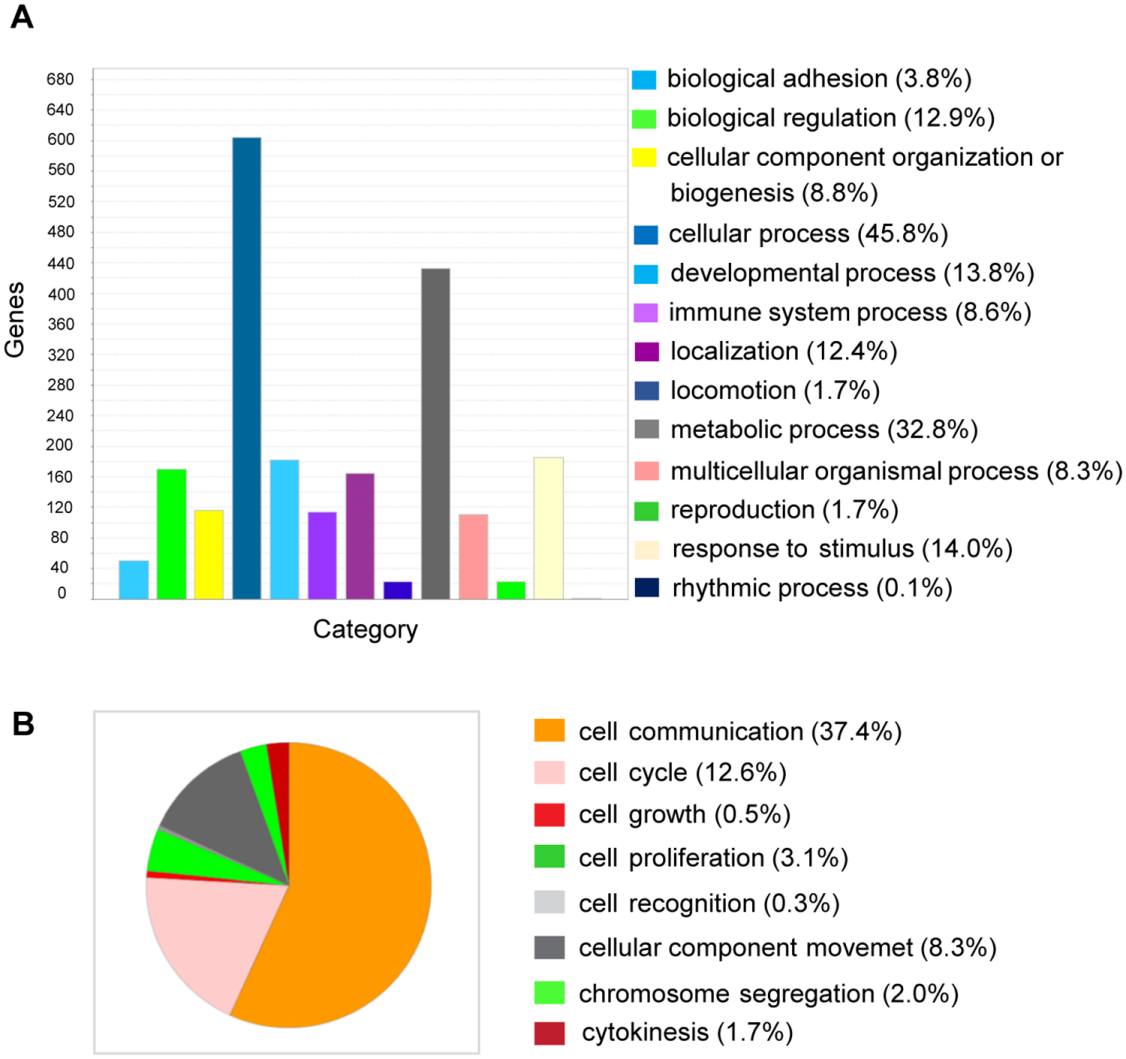
Histogram and pie chart of the GO classification of differentially expressed genes using the PANTHER Classification System. (A) The functional classification on the basis of the biological process shows an overrepresentation of differentially regulated genes involved in cellular and metabolic processes. (B) The majority of genes that are differentially regulated within the category of cellular process are involved in cell communication, cell cycle and cellular component movement. The percentages in Figs 2A and 2B correspond to the genes assigned to a specific GO term over the total number of differentially expressed genes.

The enrichment analysis of the differentially expressed genes based on GO biological process is depicted in Fig 2A. According to the GO categories, the majority of differentially regulated genes could be categorized into cellular (45.8%) and metabolic (32.8%) processes. Within the GO category cellular process, differentially regulated genes involved in cell communication are overrepresented (Fig 2B).

The MetaCore pathway analysis software (GeneGo Inc.) was used to identify the GeneGo Pathway Maps, Networks, and Processes. The analysis of both up-and downregulated genes revealed that genes of the Wnt (p-value = 1.119e-7; S2 Table) and Shh (p-value = 8.456e-5; S3 Table) signaling pathways were significantly overrepresented.

### Expression of hair cycle regulatory genes

We specifically analyzed genes of the Wnt, Shh, Bmp, Fgf and Tgf-β signaling pathways which are also known to play a role in HF biology [10]. We identified a total of 47 differentially expressed genes involved in one of these pathways (S4 Table).

**Fig 3:**
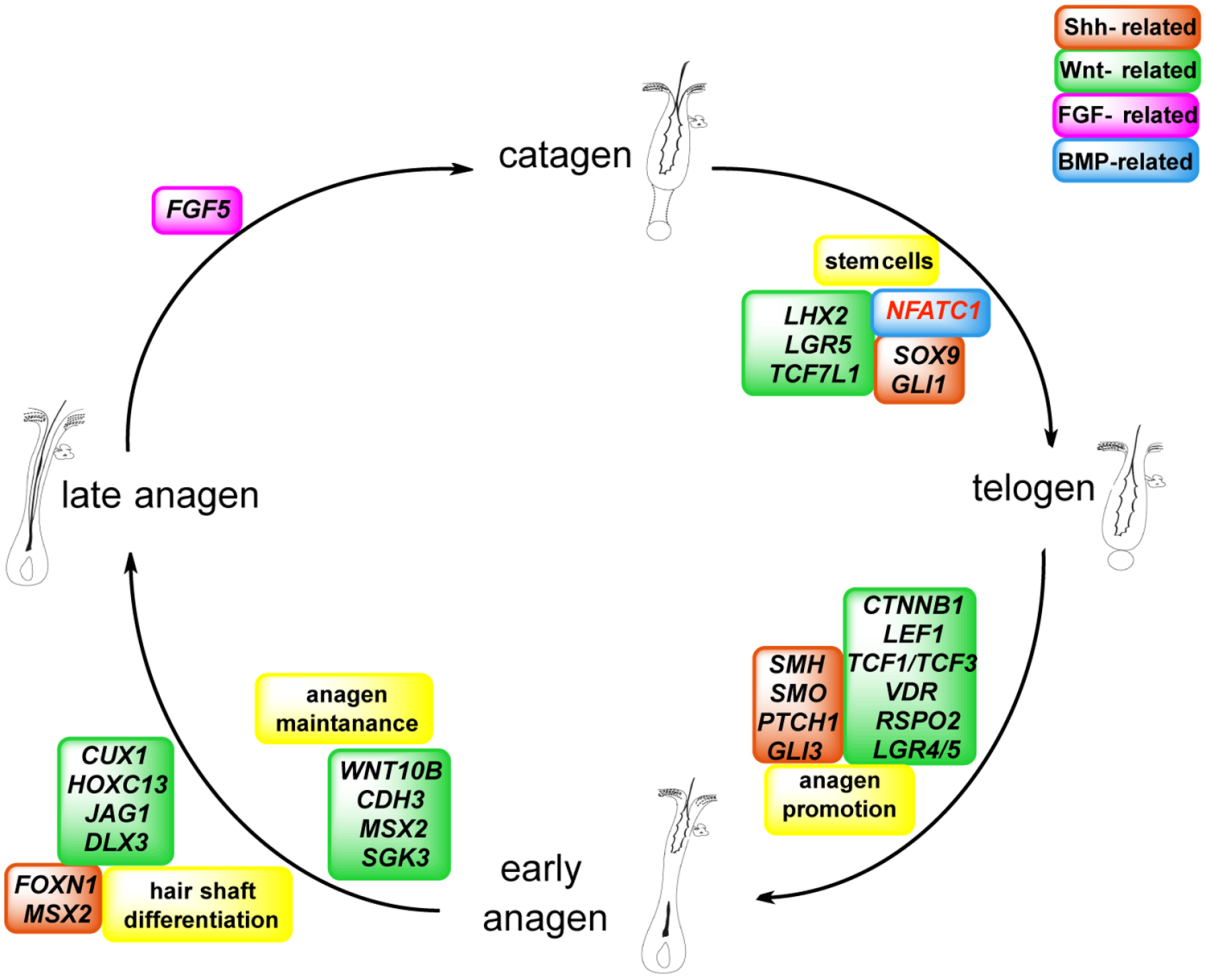
Differentially expressed genes in dogs with AX and their physiological function during specific hair cycle phases. Black indicates downregulation and red indicates upregulation of the respective genes.

The physiological role of some selected differentially regulated genes within the HC is shown in Fig 3.

### Expression of hair follicle stem cell markers

Next, we investigated the expression of genes coding for known HF SC markers [6, 7, 25–29] and found differential expression of several SC and progenitor markers. Whereas *SOX9*, *LHX2*, *LGR5*, *TCF7L1* and *GLI1* were downregulated, the transcription factor *NFATc1*, a HF SC quiescent marker, was upregulated (S1 Table, Fig 3).

### Differentially expressed genes involved in steroidogenesis, melatonin metabolism and in the vitamin D receptor pathway

**Fig 4:**
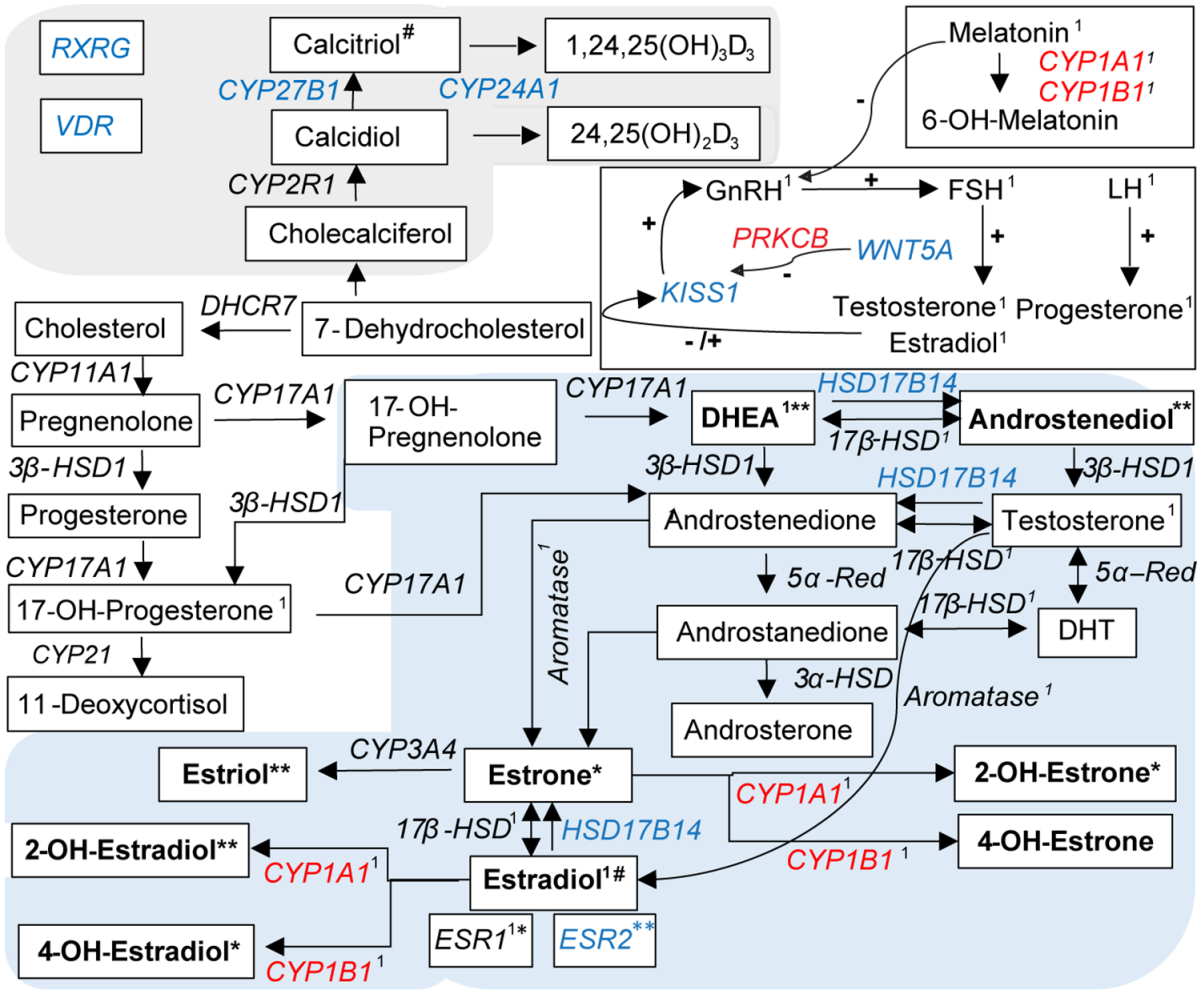
Differentially expressed genes playing a role in sex hormone biosynthesis and metabolism. Sex hormone biosynthesis and metabolism is accomplished by a complex cascade involving hormones and enzymes of the hypothalamic-pituitary-gonadal axis, the vitamin D synthesis and the pineal gland hormone melatonin. Since it has been shown that the skin has its own neuroendocrine system these complex interactions may also occur in the skin. They are depicted in Fig 4. The differentially regulated genes involved in this process are indicated in blue if they were downregulated and red if they were upregulated. Abbreviations: DHEA, dehydroepiandrosterone; DHT, dehydrotestosterone; CYP, cytochrome P 450 family enzymes; 5α-Red, 5α- Reductase; ESR, estrogen receptor; GnRH, gonadotropin- releasing hormone; FSH, follicle- stimulation hormone; LH, luteinizing hormone; *KISS1*, kisspeptin 1 gene. In bold: endogenous female sex hormones; their affinity for estrogen receptors is marked with * for higher affinity to ESR-α encoded by *ESR1* and ** for higher affinity to ESR-β encoded by *ESR2*. ^1^ indicates that melatonin decreases the levels of these. ^#^ Calcitriol increases levels of estradiol.

In addition to the differentially regulated genes involved in known signaling pathways associated with the HC we identified 10 differentially regulated genes involved in the hypothalamic-pituitary-gonadal axis including sex hormone synthesis, vitamin D synthesis and melatonin metabolism (S5 Table, Fig 4).

## Discussion

In our study we identified 47 differentially expressed genes in the alopecic skin of Pomeranians with AX that are known play a role in murine HF physiology, suggesting that the canine HC is controlled similar to mice. The majority of the differentially regulated genes codes for molecules related to the Wnt- and Shh-pathways, which are the major players for anagen induction and maintenance (reviewed in [30–34]). In our dataset we found a downregulation of genes coding for the secreted Wnt proteins 10B and 5A (*WNT10B*, *WNT5A*) as well as for the Wnt signaling receptors frizzled 3, 7, and 10 in dogs with AX. In addition our data indicate that Wnt downstream signaling is impaired since genes coding for β-catenin (*CTNNB1*) and members of the T cell factor/lymphoid enhancer factor family of transcription factors (*LEF/TCF)*, numerous other genes coding for important signaling molecules and transcription factors of the Wnt signaling pathway (e.g. *DLX3*, *CTNNB1*, *HOXC13*, *CUX1)*) as well as Wnt target genes (such as *JAG1 and CDH3)* are downregulated [35]. All of these molecules play a role in the HC. Furthermore, also genes coding for Wnt agonists such as *RSPO2* and agonist receptors such as *LGR4* and *LGR5* are significantly downregulated. R-spondins are highly expressed in mouse dermal papilla cells and are known to regulate HC progression by activating HF SCs in altering the cell fate determination but not SC proliferation [36]. Downregulation of Wnt is further supported by the upregulation of Wnt antagonists such as *WIF1* and *MMP7* [37, 38].

Furthermore, genes of the Shh pathway are downregulated, including *SHH* and *SMO*, its receptor *PTCH1*, and the transcription factors *GLI1* and *GLI 3*. It has been shown that Shh signaling is involved in anagen initiation of both primary and secondary HFs and is expressed at high levels in the matrical cells of the HF bulb and thus is involved in the promotion of anagen [39–41].

Bmp signaling has been shown to regulate the HC by inhibiting anagen induction [42, 43]. Thus, we expected an upregulation of Bmp signaling molecules in our dataset. However, unexpectedly we found a downregulation of *BMP4*, *SMAD2 and SMAD7* all known to play a role in SC quiescence [43]. Since at the same time *NFATc1*, a HF quiescence marker, occurring upstream of Bmp signaling was upregulated and a downregulation of some Bmp antagonists (e.g. *BAMBI*) is seen, we assume that the delicately balanced Bmp pathway, involving an intimate interaction of many genes and cellular events is nevertheless differentially regulated thus resulting in telogen arrest despite the downregulation of some BMP signaling molecules [44–46].

The histological findings in skin biopsies from dogs with AX which clearly show an abundance of telogen and kenogen HFs are in line with the transcriptome data suggesting an impaired anagen induction and promotion [19].

We could identify many follicular SC and progenitor markers in the canine transcriptome of healthy dogs that were also reported in mice and humans [7, 25–28]. Importantly, some of these were downregulated in dogs with AX *(TCF7L1*, *LHX2*, *LGR5*, *GLI1* and *SOX 9*). A downregulation of HF SC markers has also been described in men with androgenic alopecia [47]. However, further investigations are warranted in men and dogs to elucidate, if the downregulation of specific SC markers is specific for certain alopecic conditions or the consequence of more general changes in diseased skin.

Besides the deregulation of genes playing a role in the HC and HF SC markers, our dataset derived from skin biopsies reveals the deregulation of genes known to be relevant for the steroid hormone metabolism.

An altered steroid hormone metabolism has been suggested by other authors as underlying cause for AX but was never proven [12, 48–51]. This may partially be due to the fact that the steroid hormone metabolism was assessed in the plasma of dogs and it is well known that plasma hormonal concentrations vary substantially between individuals and within one individual [51, 52]. Furthermore, there is increasing evidence that the skin itself has powerful neuroendocrine activities and hormones are produced and metabolized locally [53–56]. Thus, measuring hormonal levels in the plasma may not be the appropriate method since a deregulation directly in the skin may be causative for the disease. Since it has been shown, in various studies that estrogen plays a major role in HF biology, specifically the inhibition of telogen-to-anagen transition [57, 58] the hypothesis that an altered sex hormone metabolism plays a role in the pathogenesis of AX deserves further investigations. Our dataset, derived from skin biopsies, supports the hypothesis that the sex hormone metabolism is locally deregulated in dogs with AX. We observed a significant downregulation of *HSD17B14* which codes for the 17β-hydroxysteroid dehydrogenase14 (17β-HSD14) and an upregulation of the cytochrome P450 family members *CYP1A1* and *CYP1B1* as well as the downregulation of the estrogen receptor (*ESR*)*1*. It has been shown that 17β-HSD14 is expressed in the skin and that it oxidizes estradiol into the less bioactive steroid metabolites estrone (Fig 4) [59]. The upregulation of *CYP1A1* and *CYP1B1*, involved in metabolizing estradiol and estrone to less bioactive metabolites further supports the hypothesis that the estrogen metabolism is differentially regulated in dogs with AX. In addition, the gene coding for *ESR2* but not *ESR1* is downregulated in our data set. The affinity of ligands to ESRs differs. For example, the short-acting 17α-estradiol has a higher affinity to ESR-α encoded by *ESR1* whereas the biologically weak estriol has a higher affinity to the ESR-β encoded by *ESR2* [60]. Elevation of estriol concentrations as a consequence of augmented estrone after downregulation of *HSD17B14* could therefore explain the downregulation of *ESR2* and not *ESR1* that we see in our dataset. The fact that *ESR1* encoding for ESR-α is not downregulated in our data set is also supported by a study in which the estrogen receptor staining in the hair follicles was assessed in dogs with AX treated with melatonin and hair regrowth was not associated with a change in staining intensity [49].

Another strong indication to support the hypothesis that the estrogen metabolism is differentially regulated is that the vitamin D receptor gene (*VDR)* and the Retinoid X Receptor Gamma gene (*RXRG*, whose protein forms heterodimers with VDR), as well as two members of the vitamin D pathway (namely *CYP27B1*, *CYP24A1)* are significantly downregulated. *CYP27B1*, encodes for an enzyme which activates Vitamin D3 and *CYP24A1* is involved in Vitamin D degradation (Fig 4). Furthermore Vitamin D has been shown to play an essential role in the biosynthesis of estradiol in mice and pigs [61]. It is well known that keratinocytes are the primary source of Vitamin D and its active metabolite is processed in the skin, supporting a local deregulation of the estrogen metabolism partially mediated by Vitamin D [62]. In line with a differentially regulated estrogen metabolism is also the observed treatment response of AX affected Pomeranians to melatonin [51]. The anti-estrogenic actions of melatonin are extensively reviewed [63–68]. Since melatonin is also produced and metabolized in the skin it can be assumed that *CYP1A1* and *CYP1B1*, which are known to be involved in the degradation of plasma melatonin, are also involved in the degradation of melatonin in the skin and treatment of dogs with AX with melatonin at least partially compensates for this local degradation [69–71]. Interesting in the context of a deregulated sex hormone metabolism is also the downregulation of the gene coding for kisspeptin (*KISS1*) in our data set. The downregulation of *kisspeptin* is known to result in a lower GnRH release in the arcuate nucleus which in turn has via hypothalamo–pituitary–adrenal axis an effect on the sex hormone metabolism [72]. It has been shown that within the skin an own neuroendocrine system exists which functions similar to the hypothalamo–pituitary–adrenal axis, catecholaminergic, cholinergic, steroidogenic and secosteroidogenic systems [73–75]. Since we found a downregulation of kisspeptin in the skin we can speculate that kisspeptin is also effective in the local endocrine metabolism. If this local deregulation is the consequence of a systemic deregulation or occurs only in the skin warrants further investigations. It has been reported that after the implantation of deslorelin, a GnRH analogue hair was regrowing in 75% of the intact male Pomeranians with AX and there was an 60% overall positive response if neutered female Pomeranians with AX were included [50]. The positive effect of the treatment with a GnRH analogue supports that the GnRH expression may be lower in dogs with AX. The fact that treatment with melatonin, which negatively regulates GnRH secretion, resulted in hair regrowth in about 60% of the AX affected dogs, however indicates that the regulatory mechanisms are more complex than known to date [51].

In summary, our results provide novel insights into the molecular factors that govern the canine HC and their disruption in AX, which has not been shown before. They show that the canine HC is regulated by similar signaling pathways as in mice. In addition, we show a deregulation of genes maintaining the SC compartment and the HC in AX dogs. If the downregulation of some of the SC markers is specific for AX or is a consequence of alopecia in general needs to be investigated by comparing our dataset with datasets from dogs with other non-inflammatory alopecic disorders. Furthermore, our data strongly support the previously established hypothesis that the steroid hormone metabolism is altered in Pomeranians with AX. More specifically, there are indications that the estrogen metabolism is differentially regulated in the skin.

## Supporting information

S1 Table Results of differential expression analysis. Significantly differentially expressed genes with a false discovery rate (FDR) ≤ 0.01, sorted by log2 Fold Change; BaseMean, mean of normalized read counts across all samples; LfcSE, standard error of the log2FoldChange; Stat, the log2FoldChange divided by lfcSE.
(XLSX)

S2 Table Wnt-related genes. BaseMean, mean of normalized read counts across all samples; Log2FC, log2 fold change; FDR, False discovery rate, refers to Benjamini-Hochberg adjusted p- value.
(XLSX)

S3 Table Shh-related genes. BaseMean, mean of normalized read counts across all samples; Log2FC, log2 fold change; FDR, False discovery rate, refers to Benjamini-Hochberg adjusted p- value.
(XLSX)

S4 Table Differentially expressed genes known to be involved in the murine HC in alopecic skin from dogs with AX as compared to healthy Pomeranians. BaseMean, mean of normalized read counts across all samples; Log2FC, log2 fold change; FDR, False discovery rate, refers to Benjamini-Hochberg adjusted p- value.
(XLSX)

S5 Table Differentially expressed genes involved in the regulation of gonadotropin releasing hormone and sex hormone synthesis, vitamin D synthesis and melatonin metabolism. BaseMean, mean of normalized read counts across all samples; Log2FC, log2 fold change; FDR, False discovery rate, refers to Benjamini-Hochberg adjusted p- value; HF, hair follicle; GnRH, Gonadotropin-releasing hormone; ORS, outer root sheath; DP, dermal papilla.
(XLSX)
